# A Sinister Bias for Calling Fouls in Soccer

**DOI:** 10.1371/journal.pone.0011667

**Published:** 2010-07-07

**Authors:** Alexander Kranjec, Matthew Lehet, Bianca Bromberger, Anjan Chatterjee

**Affiliations:** Neurology Department and the Center for Cognitive Neuroscience, University of Pennsylvania, Philadelphia, Pennsylvania, United States of America; University College London, United Kingdom

## Abstract

Distinguishing between a fair and unfair tackle in soccer can be difficult. For referees, choosing to call a foul often requires a decision despite some level of ambiguity. We were interested in whether a well documented perceptual-motor bias associated with reading direction influenced foul judgments. Prior studies have shown that readers of left-to-right languages tend to think of prototypical events as unfolding concordantly, from left-to-right in space. It follows that events moving from right-to-left should be perceived as atypical and relatively debased. In an experiment using a go/no-go task and photographs taken from real games, participants made more foul calls for pictures depicting left-moving events compared to pictures depicting right-moving events. These data suggest that two referees watching the same play from distinct vantage points may be differentially predisposed to call a foul.

## Introduction

For soccer referees, deciding whether to call a foul often means making a quick judgment about a fast-moving, dynamic event. Seeing a foul depends greatly on context and timing. The perception of contact is not enough to distinguish between a fair and unfair tackle. Even post-match expert analyses using slow-motion replays can end equivocally. The ambiguous nature of soccer fouls becomes especially relevant when a single call dramatically alters the course of a game. This situation is perhaps more true for soccer compared to other sports. In soccer, fouls result in free kicks; free kicks frequently lead to goals; and a single goal is often the difference between a winning and losing team. During a competition like the World Cup the stakes are especially high. Success can affect the economies and politics of nations. Enraged fans have been known to react violently to the perceived mistakes made by individual players and referees [1].

Low-level perceptual biases can influence higher-order officiating judgments in other sports that involve ambiguity [2]. We wondered if soccer foul judgments could be modulated by a well documented perceptual-motor bias. Readers of left-to-right written languages tend to conceptualize events as traversing space concordantly. Although data suggests that brains are wired with some default preferences for left-to-right motion, there is enough evidence in populations that read right-to-left languages (e.g. Hebrew and Arabic) to conclude that perceptual and motor habits associated with the development of literacy influence how we think about canonical representations of events [3]–[6]. Thus readers of left-to-right languages are more likely to put a circle on the left when asked to draw a simple event like “the circle pushes the square” [7] and to rate goals scored from left-to-right as more beautiful than goals scored in the opposite direction [8]. This phenomenon is exploited by filmmakers who both regularly depict protagonists entering from *screen left—moving right*, and invert the same principle for antagonists, having them enter from *screen right—moving left*; the idea being that our discomfort with leftward motion will transfer onto the bad guy [9]. According to Hollywood folklore, a similar technique was used extensively in *Apocalypse Now* [10]. Presumably to disturb viewers, the bulk of travel along the river into the jungle moves leftward.

Given this bias for representing prototypical events from left-to-right, English speakers should be more likely to call a foul when the direction of play moves leftward. Below awareness, left-moving events should seem atypical and relatively debased compared to right-moving events. For soccer-knowledgeable participants making refereeing judgments in ambiguous situations, this perceptual-motor bias may serve to lower the threshold for calling a foul.

## Methods

### Ethics Statement

This study was approved by the Institutional Review Board at the University of Pennsylvania. The work was conducted according to the principles expressed in the Declaration of Helsinki. Written informed consent was obtained from all participants.

### Participants

We recruited twelve right-handed (and right-footed) members of the University of Pennsylvania's varsity soccer teams (4 males, 8 females; mean age  = 19.3 years). All were native English speakers. Players were paid in return for their participation. Each completed a 12-item questionnaire assessing the extent of their playing and watching experience, and their knowledge of soccer rules, professional teams and players.

### Stimuli

All photos used for stimuli depicted scenes from English Northern League or Premiership reserve team matches. Both leagues are obscure by American standards so participants were unlikely to be familiar with individual players. Initially, photographs were chosen according to three criteria. Selected photos depicted (1) scenes with only 2 athletes directly involved, where (2) one was clearly the player with the ball, while the other attempted to disrupt possession, and lastly, (3) a strong implied rightward or leftward direction of movement. Using Photoshop, numbers and letters were removed from uniforms and backgrounds, photographs were resized to common dimensions (500×357 pixels), and flipped along the x-axis to create left-moving and right-moving versions.

Prior work has demonstrated that static photographs depiciting implied motion can evoke perceptual and neural effects associated with motion processing [11], [12]. We carried out a norming study to verify the particular directionality implicit in each photo and to ensure that information about directionality could be extracted from the stimuli over 500 ms presentations. Stimuli were presented on a laptop computer. Participants pressed the left arrow key if the picture depicted leftward motion, and the right arrow key if it depicted rightward motion. Of the over 200 normed pairs, the best 134 pairs (268 total pictures including the original and flipped versions) rating above 85% agreement for directionality were selected for use in the experiment. We found a significant reaction time difference in assigning direction for rightward (693.8 ms) vs. leftward (746.1 ms) photographs, *t*(5,1) = 4.12, p<0.01. This demonstrates that on a task that explicitly requires attending to motion, these stimuli elicited reaction time differences consistent with a left-right bias; photographs depicting rightward motion were processed more quickly, at least when participants were thinking about directionality.

### Procedure

In an actual game, referees must decide to either make a foul call or not. Accordingly, we used a go/no-go task, as such a task would require participants to quickly make a meaningful judgment and decide to either make or withhold a response (i.e. a foul call), much like a referee must do in a real game. Participants were informed that they would view a number of confrontations between attacking and defending players on a laptop computer and that they should press the spacebar if the defending player committed a foul but make no response if no foul was committed. There were 20 practice trials and 268 experimental trials. A trial consisted of a 2000 ms fixation cross, then a picture presented for 500 ms followed by a 3000 ms response screen. Each participant saw all 134 pictures in both orientations. Pictures were presented pseudorandomly such that, when a particular stimulus in one orientation was randomly presented in the first half of the experiment, its counterpart was presented in the second half. This step was taken to minimize the possiblity of participants becoming aware of the orientation manipulation. Participants responded to 50% of trials with their right hands and 50% with their left.

## Results

**Figure 1:**
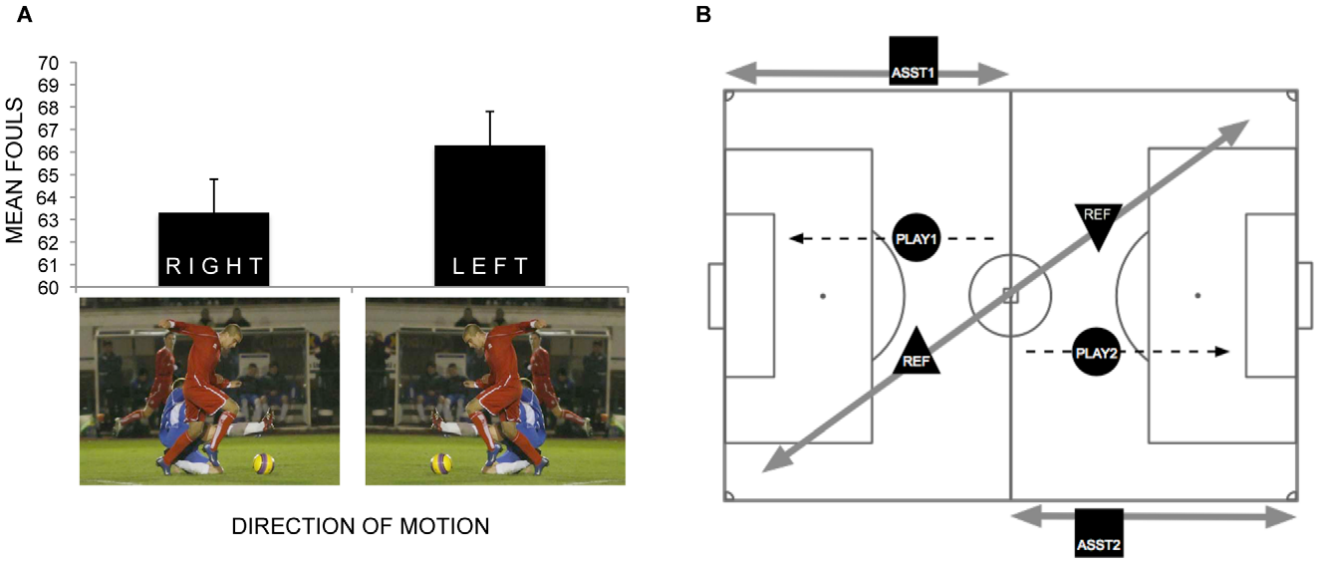
Average number of fouls called with two example stimuli and the standard left diagonal system of control. (A) The mean number of fouls called is greater for left-moving pictures. (B) In the left diagonal system, the referee (REF) will generally observe a play (PLAY1 & 2) unfold from right-to-left in the attacking third of the field. For the assistants (ASST1 & 2), attacking play will always unfold from left to right. These opposing perspectives should lead to referees having a lower threshold for making fouls calls relative to assistants in the attacking half of the field.

On average, participants called aproximately 3 more fouls when pictures were viewed in their left-moving (66.5 fouls) compared to their right-moving (63.3 fouls) orientation. A paired sample t-test found this difference to be significant *t*(11) = 2.21, *p*<.05. Although not large (Cohen's *d* = 0.18), these effects are impressive considering that *within subjects* the pictures for each trial type (left-moving vs. right-moving) were the same in every respect other than the direction of implied motion. This finding means that an individual participant was more likely to call a foul when seeing a picture in its leftward compared to rightward orientation, even though the two stimuli were otherwise identical. (See Figure 1A.)

Participants were no faster in making responses to leftward or rightward stimuli. A paired sample t-test found no reaction time differences for direction (p>0.30). Also, the hand used by participants did not have a significant effect on response times. Neither a paired t-test performed on the average number of fouls called with each hand, nor a 2×2 ANOVA testing for a hand x direction interaction were significant (p's>0.60).

Questionnaire data was collected. Answers to 4 agreement rating questions using a 5-point Likert scale demonstrated that this group of elite athletes had considerable playing and watching experience (e.g., “I have played a lot of organized league soccer”: mean agreement rating  = 5.00; “I have watched many soccer games on television”: mean agreement rating  = 4.33). Performance on 8 multiple-choice questions confirmed that participants possessed a basic knowledge for the official rules of the game (e.g. “How far is the penalty spot from the goal line?”: average % correct  = 100) and for facts pertaining to professional teams and players (e.g. “Liverpool FC traditionally wears which color at home?”: average % correct  = 91.67).

## Discussion

The present study found that soccer-knowledgable participants were more likely to call fouls when pictures of player confrontations depicted leftward compared to rightward motion. Although we cannot draw strong conclusions regarding the mechanism underlying this result, the effects are consistent with the idea that our population's familiarity with the left-right organization of writing and other cultural artifacts—like comics, calendars, and diagrams—affect the interpretation of ambiguous stimuli. In discussing the results of a related study's finding that Italian speakers rated soccer goals scored from left-to-right as more beautiful than goals scored from right-to-left, and the opposite pattern for speakers of Arabic, Maass and colleagues refer to “a subjective feeling of fluency” that can influence higher-level judgments [8]. The present results suggest that perceptual fluency can affect negative as well as positive judgments.

How might these perceptual biases play out on the soccer field? From the vantage point of a professional referee using a recommended officiating system for international matches, *actual* left-to-right or right-to-left directional regularities are more inscribed onto the general run of play than one might expect. That is, compared to the participants in the current study who saw equal numbers of plays moving in both directions, officials working real matches are likely exposed to a more restricted set of either left-moving or right-moving attacking plays depending on the role they occupy in a particular system. Why should this be the case?

The standard *diagonal system of control* recommends that referees patrol the diagonal between corner flags, while assistants run the two opposite touchlines. When Sir Stanley Rous (president, FIFA, 1961-1974) codified the diagonal system into the Laws of the Game [13], it was probably with the belief that providing two observers on opposite sides of every play could only serve to decrease ambiguity on the field. However, if seeing a play unfold from right-to-left really does lower the threshold for calling a foul, it could mean several things for officials using a standard system.

The most obvious point is that the referee and assistants will generally have conflicting pespectives with respect to left-right motion. In the more common *left diagonal system*, both teams' attacking runs on goal in their respective offensive thirds of the field—where called fouls can be very valuable—will usually be moving leftward for referees and always be moving rightward for assistants (Figure 1B). This means that if the spatial biases we observed in our population of soccer players have the same effects on referees in real matches, they should influence particular officials differently: referees on the field will more frequently be in a position that should afford a lower relative threshold for calling fouls during an attack compared to assistant referees working the lines. The particular diagonal a referee chooses to use is also relevant. The results of the present study suggest that the use of the left diagonal system should favor the offense for both teams, as the referee would be expected to be more predisposed to call fouls during attacking plays at both ends. Conversely, a *right diagonal system* of control should favor the defense for both teams, as the referee will generally observe attacks on goal unfold from left-to-right and therefore be expected to call fewer fouls. These relational oppositions suggest that referees should avoid changing diagonals at halftime if possible. According to the current Laws, there is nothing to prevent referees from switching from right to left diagonals (or vice versa) at halftime in order to account for changing light conditions or other concerns. For example, switching from a left-to-right diagonal at halftime, which the current model suggests would favor defensive play in the second half for both teams, could provide an unfair advantage to a team with a lead. An analysis of match data is needed to determine if more fouls are in fact called in the attacking thirds of the field when referees use a left compared to right diagonal system of control.

Yet when referees run diagonals consistently during both halves, left-right directional regularities are the same for both teams. So soccer may have stumbled onto a good system, at least in the context of the present study's focus. With respect to the spatial biases we report here, the diagonal system of control is better than older linear systems that required referees to patrol a straight path along a single touchline. The use of a linear system results in a left-moving attack for one team and a right-moving attack for another. This would in principle provide each team with an attacking advantage for the duration of a half.

Regardless of the system employed, a referee will see plays unfold in both right-to-left and left-to-right directions; use of a diagonal system only constrains directional biases. Future research is needed to determine if left-right directional biases exert more or less influence on the field. Since the current study used a population of soccer players viewing static photographs, rather than professional referees in a more realistic setting, one can only make inferences regarding the degree to which these effects may apply in game conditions. However, it is possible that extant match statistics could be analyzed to determine if left-right spatial biases influence the number of fouls called with respect to the hypotheses generated by the present experimental data. Furthermore, in order to conclude with any certainty that the effects we report result from reading habits, other populations (e.g. Arabic or Hebrew readers) will need to be tested directly. Although previous studies [5], [6] have found evidence to suggest that similar directional effects are reversed in populations that read left-right languages, we acknowledge that we did not test this hypothesis directly in this study. Regardless, these results at least suggest that the effects of low-level perceptual mechanisms could alter a decision, change the result of a game and perhaps, the fortunes of nations.
